# Salinity tolerance, Na^+^ exclusion and allele mining of *HKT1;5* in *Oryza sativa* and *O. glaberrima*: many sources, many genes, one mechanism?

**DOI:** 10.1186/1471-2229-13-32

**Published:** 2013-02-27

**Authors:** John Damien Platten, James A Egdane, Abdelbagi M Ismail

**Affiliations:** 1 International Rice Research Institute, Los Baños, Philippines

**Keywords:** Allele mining, HKT, Mechanisms of salt tolerance, Rice, Salinity

## Abstract

**Background:**

Cultivated rice species (*Oryza sativa* L. and *O. glaberrima* Steud.) are generally considered among the crop species most sensitive to salt stress. A handful of lines are known to be tolerant, and a small number of these have been used extensively as donors in breeding programs. However, these donors use many of the same genes and physiological mechanisms to confer tolerance. Little information is available on the diversity of mechanisms used by these species to cope with salt stress, and there is a strong need to identify varieties displaying additional physiological and/or genetic mechanisms to confer higher tolerance.

**Results:**

Here we present data on 103 accessions from *O. sativa* and 12 accessions from *O. glaberrima*, many of which are identified as salt tolerant for the first time, showing moderate to high tolerance of high salinity. The correlation of salinity-induced senescence (as judged by the Standard Evaluation System for Rice, or SES, score) with whole-plant and leaf blade Na^+^ concentrations was high across nearly all accessions, and was almost identical in both *O. sativa* and *O. glaberrima*. The association of leaf Na^+^ concentrations with cultivar-groups was very weak, but association with the *OsHKT1;5* allele was generally strong. Seven major and three minor alleles of *OsHKT1;5* were identified, and their comparisons with the leaf Na^+^ concentration showed that the *Aromatic* allele conferred the highest exclusion and the *Japonica* allele the least. A number of exceptions to this association with the *Oryza HKT1;5* allele were identified; these probably indicate the existence of additional highly effective exclusion mechanisms. In addition, two landraces were identified, one from Thailand and the other from Senegal, that show high tissue tolerance.

**Conclusions:**

Significant variation in salinity tolerance exists within both cultivated *Oryza* species, and this is the first report of significant tolerance in *O. glaberrima*. The majority of accessions display a strong quantitative relationship between tolerance and leaf blade Na^+^ concentration, and thus the major tolerance mechanisms found in these species are those contributing to limiting sodium uptake and accumulation in active leaves. However, there appears to be genetic variation for several mechanisms that affect leaf Na^+^ concentration, and rare cases of accessions displaying different mechanisms also occur. These mechanisms show great promise for improving salt tolerance in rice over that available from current donors.

## Background

The development of improved rice varieties with high tolerance of salt stress has been a major and long-standing goal of rice breeding efforts. Salinity is a significant constraint to rice productivity in many inland and coastal rice-growing areas and, furthermore, is contributing to the loss of arable lands in many countries due to salt accumulation as a result of excessive use of irrigation water with poor or improper drainage, a fact that is likely to be aggravated by sea level rise in coastal areas caused by climate change [1-3]. Therefore, efforts to improve the salinity tolerance of rice and many other crops are intensifying. Significant bodies of work have been accomplished on the characterisation of physiological responses affected by salt stress. These studies highlighted the complexity of the mechanisms involved in rice in which tolerance varies with the stage of development, with the crop being relatively more tolerant during germination and active tillering as well as during late grain filling and maturity, but sensitive during the early vegetative and reproductive stages [4,5], and with weak association between the degree of tolerance at the two sensitive stages [6].

Numerous physiological studies on the mechanisms of tolerance during the vegetative stage have been published [5,7-9], most of which showed an inverse relation between shoot Na^+^ content and/or Na^+^/K^+^ ratio and plant survival, injury scores and grain yield [10,11]. Other traits suggested to be associated with salt tolerance in various studies are compartmentation of Na^+^ in older leaves and leaf sheaths and in the vacuoles, maintenance of mineral nutrient homeostasis, especially K^+^ and Ca^2+^, high selectivity for K^+^ and/or Ca^2+^ uptake over that of Na^+^, limiting effects of reactive oxygen species (ROS scavenging), accumulation of compatible solutes to offset osmotic effects (osmotic adjustment), maintenance of leaf area index and maintenance of tiller number [5,9-13]. The importance of the apoplastic bypass flow in delivering Na^+^ to the xylem, thus reducing leaf Na^+^ concentration and improving tolerance, has also been noted [14-17]. During the reproductive stage, tolerant genotypes strongly exclude salt from flag leaves and developing panicles [6,18]. The complexity of tolerance highlighted in these studies suggests the need for combining tolerance mechanisms at each stage as well as at the two most sensitive stages to develop varieties that are widely adapted to salt-affected areas.

Efforts also focused on the mapping of QTL loci controlling these various component traits, and a few major loci and numerous minor loci controlling various aspects related to salinity tolerance were subsequently identified. The best known and seemingly most robust QTL is *Saltol/SKC1* on the short arm of chromosome 1 [19,20]. QTLs have been identified in this region in a number of populations derived from several donors ([21-23], A. Ismail unpublished results), and the gene has been identified to a high degree of confidence as *OsHKT1;5*[24] (see [25] for nomenclatural clarification). A very recent association mapping effort using varieties from the japonica cultivar-group [21] has also identified the *Saltol* genomic region as controlling important aspects of salinity tolerance, as well as validating many other QTLs. In wheat, two members of the *HKT* gene family (including the wheat *HKT1;5* orthologue) have also been shown to co-localise with major QTLs [26-28], and the *HvCBL4* gene, a homologue of the *Arabidopsis SOS3* known to confer salt tolerance, mapped to a genomic region similar to that of a barley salt tolerance QTL [29].

In addition to the *Saltol* locus, many other QTLs have been identified in rice (e.g. see [8] for a recent review). Several of these appear to be common among multiple mapping populations, though they seem to be derived from the same or genetically similar donors. Examples include the long arms of chromosomes 1, 3 and 6 [20,22,30-35]. Although numerous studies have identified hundreds of genes involved in salt stress responses, many of which lead to improved tolerance when over-/underexpressed, and some co-localise with QTL regions, studies on the cloning of other QTLs in cereals are yet to be published. This might be in part due to these additional QTLs typically controlling much smaller portions of the total variance than does *Saltol*, and thus being more difficult to work with.

Despite the long history of salinity tolerance research and breeding efforts, very few large-scale screening efforts have been undertaken. A number of authors published studies involving small numbers of accessions [10,36-41]. Only four studies appear to have examined more than a few dozen accessions [9,42-44], and even these have focused on breeding lines and improved cultivars, which tend to stem from a small donor pool typically involving Pokkali and/or Nona Bokra as donors. In screens of 21 and 38 genotypes of wheat (*Triticum aestivum* L.) [45], the authors concluded that leaf Na^+^ concentration showed little correlation with performance, and that Na^+^ exclusion and tissue tolerance were equally important, and segregating independently. However, even in this case, the germplasm examined consisted of breeding lines and improved cultivars stemming from a very restricted geographic area.

Thus, there seems to be a lack of large-scale screening efforts specifically aimed at identifying significant new donor germplasm, particularly with regard to traditional varieties/landraces. It would thus appear difficult to generalise about the level of salinity tolerance displayed or the mechanisms possessed by these species as a whole, factors that are important when considering breeding approaches. In addition, a common feature of nearly all these studies is screening under relatively mild salt stress, typically of electrical conductivity (EC) of 6 dS m^-1^ (approx. 50 mM NaCl) to 12 dS m^-1^ (approx. 100 mM NaCl), and there seems to be a lack of screening efforts specifically aimed at identifying highly tolerant germplasm that might therefore contain additional major QTLs as effective as or more effective than *Saltol*. The objectives of this work are to (1) screen known and novel germplasm under high salinity to identify new highly tolerant lines, with particular emphasis on traditional landraces that may have novel alleles/mechanisms of tolerance unrelated to Pokkali and Nona Bokra; (2) characterize the tolerant lines, specifically with reference to Na^+^ and K^+^ uptake, to classify lines based on tolerance mechanisms; (3) supplement phenotyping results with allele mining of the *OsHKT1;5* gene, and relate alleles to function; and (4) integrate this information with respect to *Saltol*/*OsHKT1;5* activity, and identify novel donors for use in breeding.

## Results

### Screening of diverse landraces

**Figure 1:**
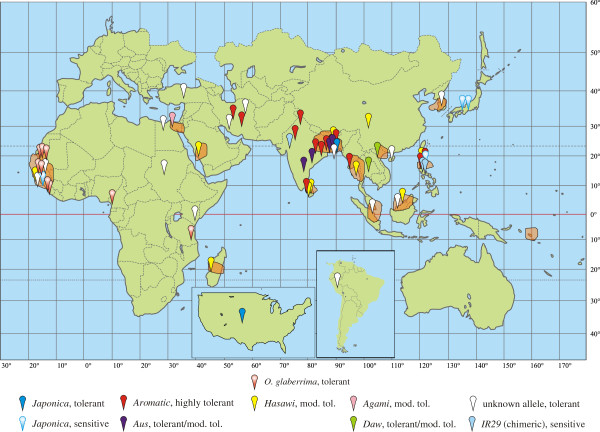
**Geographic provenance of tolerant landraces.** Geographic provenance of tolerant landraces identified in the literature or through this study, and association with *HKT1;5* allele.

Screening of approximately 550 accessions from the T.T. Chang Genetic Resources Centre of IRRI, chosen for having plausible likelihood of salinity tolerance based on origin and other passport information, resulted in the identification of 103 moderately to highly tolerant accessions, including 12 from *O. glaberrima* (Table 1 and Additional file 1: Table S1). These accessions were from diverse geographic locations, and likely span the entire geographic range of *O. sativa* (Figure 1). Some clusters of tolerance can be made out, such as those from the well-known origins of many lines in southeast India and southern Bangladesh. In addition, a number of tolerant lines were identified from regions such as Guinea/Guinea-Bissau in West Africa, Iran and the Philippines. Further examination of additional accessions from these areas may yield additional tolerant lines.

**Table 1:** Salt-tolerant accessions identified in this study

**IRGC #**	**Genotype**	**Species**	**Origin**	**Accession status**	**SES**	**Tolerance**
104022		*O. glaberrima*	Guinea-Bissau	Landrace/traditional cultivar	2.22	High
104023		*O. glaberrima*	Guinea-Bissau	Landrace/traditional cultivar	2.40	High
103459		*O. glaberrima*	Senegal	Landrace/traditional cultivar	3.00	High, segregating
103462		*O. glaberrima*	Senegal	Landrace/traditional cultivar	3.78	High
	Kalarata	*O. sativa*	India	Landrace/traditional cultivar	2.17	High
22710	Nona Bokra	*O. sativa*	India		2.17	High
108921	Pokkali	*O. sativa*	India	Landrace/traditional cultivar	2.17	High
26869	Pokkali (8558)	*O. sativa*	Sri Lanka		2.17	High
	Capsule	*O. sativa*	Bangladesh	Landrace/traditional cultivar	2.22	High
	Kutipatnai	*O. sativa*	Bangladesh	Landrace/traditional cultivar	2.22	High
	Cheriviruppu	*O. sativa*	India	Landrace/traditional cultivar	2.33	High
44131	Daw Hawm	*O. sativa*	Thailand		2.50	Very high
40593	Ching-Tai-Chan	*O. sativa*	China		2.56	High
44442	Gundang	*O. sativa*	Philippines	Landrace/traditional cultivar	2.58	High
44480	Jumbo-Jet	*O. sativa*	Philippines	Landrace/traditional cultivar	2.67	High
26577	Bora Dudh Kalam	*O. sativa*	Bangladesh	Landrace/traditional cultivar	2.78	High
37104	Hoglapata	*O. sativa*	Bangladesh	Landrace/traditional cultivar	2.78	High
32315	Mulai	*O. sativa*	Iran	Landrace/traditional cultivar	2.78	High
88396	Urichadra	*O. sativa*	Bangladesh	Landrace/traditional cultivar	2.80	Very high
26633	Gurdoi	*O. sativa*	Bangladesh	Landrace/traditional cultivar	2.83	Very high
26596	Demshi	*O. sativa*	Bangladesh	Landrace/traditional cultivar	2.89	Very high
26622	Gia Dhan	*O. sativa*	Bangladesh	Landrace/traditional cultivar	2.89	High
53637	Basmati 217	*O. sativa*	India		3.00	High
39185	BPI RI-2	*O. sativa*	Philippines	Released/improved/advanced cultivar	3.00	Very high
26602	Dharga Sail	*O. sativa*	Bangladesh	Landrace/traditional cultivar	3.00	High
15800	Eratio	*O. sativa*	Senegal		3.00	High
26615	Gachia	*O. sativa*	Bangladesh	Landrace/traditional cultivar	3.00	High
117275	Pokkali	*O. sativa*	India		3.00	Very high
37108	Horkocha	*O. sativa*	Bangladesh	Landrace/traditional cultivar	3.10	High
32281	Anbarloo Sadri	*O. sativa*	Iran	Landrace/traditional cultivar	3.11	High
3214	Celtik Tosya	*O. sativa*	Turkey		3.11	High
32311	Hassan Tareme	*O. sativa*	Iran	Landrace/traditional cultivar	3.11	High
56752	Som	*O. sativa*	Guinea-Bissau	Landrace/traditional cultivar	3.11	High
	FL478	*O. sativa*	Philippines	Breeding line	3.13	High
12880	Dom Sofid	*O. sativa*	Iran	Landrace/traditional cultivar	3.17	High
32312	Larome	*O. sativa*	Iran	Landrace/traditional cultivar	3.22	High
32313	Massan Mulat	*O. sativa*	Iran	Landrace/traditional cultivar	3.22	High
26595	Choia Mora	*O. sativa*	Bangladesh	Landrace/traditional cultivar	3.33	High
83125	Maroantrano	*O. sativa*	Madagascar	Landrace/traditional cultivar	3.33	High
77210	Rayada	*O. sativa*	Bangladesh	Landrace/traditional cultivar	3.33	High
17038	Damodar	*O. sativa*	India	Released/improved/advanced cultivar	3.44	High
6144	FR13A	*O. sativa*	India		3.50	High
56445	Walimbo	*O. sativa*	Senegal	Landrace/traditional cultivar	3.50	High
26576	Bora Dhan	*O. sativa*	Bangladesh	Landrace/traditional cultivar	3.67	High
16817	Hasawi	*O. sativa*	Saudi Arabia		3.67	High
4154	Taangteikpan	*O. sativa*	Myanmar		3.67	High
70635	Msalim Jaro	*O. sativa*	Kenya	Landrace/traditional cultivar	3.70	High
3401	Carolina Seln	*O. sativa*	Peru	Landrace/traditional cultivar	3.75	High
16767	Ta Lay	*O. sativa*	Vietnam	Landrace/traditional cultivar	3.83	High
1723	Carolina Gold	*O. sativa*	United States		3.89	High
49051	Rajasail	*O. sativa*	Bangladesh	Landrace/traditional cultivar	3.89	High
43287	ARC 18567	*O. sativa*	India		4.00	High
26594	Chini Sokkor	*O. sativa*	Bangladesh	Landrace/traditional cultivar	4.00	High
117282	Cypress	*O. sativa*	United States	Released/improved/advanced cultivar	4.00	High

Selected tolerant accessions identified and/or examined in this study. Passport information is derived from annotation in the T. T. Chang Genetic Resources Centre database.

**Figure 2:**
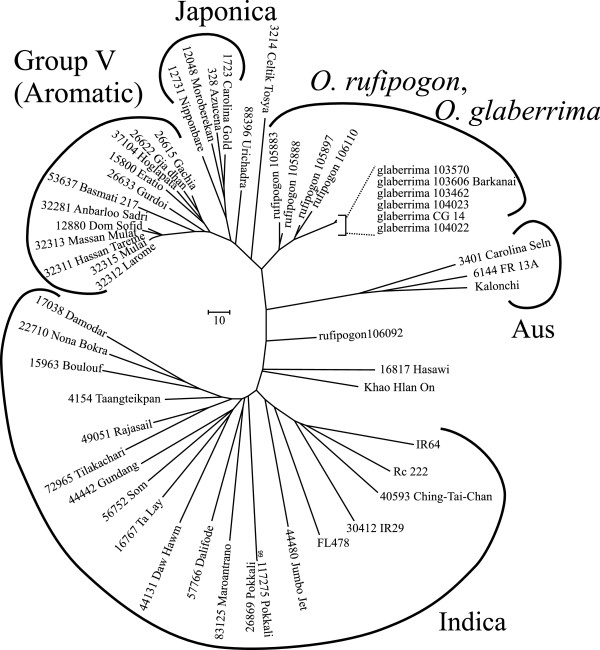
**Tolerant landraces stem from all cultivar-groups of *O. sativa.*** SNP genotyping on the 384-plex indica-indica Illumina set [46]. The majority of tolerant lines identified fall within the indica cultivar-group, but a large number originate from the aromatic cultivar-group, and other cultivar-groups are also represented. Additional lines found to be tolerant and known to be in particular cultivar-groups are listed by the indicated clades.

Likewise, the lines identified were genetically and phenotypically diverse. SNP genotyping of selected lines showed that while many were from the indica cultivar-group, as is often presumed, a very significant number also came from the group V (aromatic) cultivars (Figure 2). In addition, several accessions from the aus and tropical japonica clades were identified that show significant tolerance.

### Correlation of SES scores and concentrations of Na^+^ and K^+^ in plant tissue

**Figure 3:**
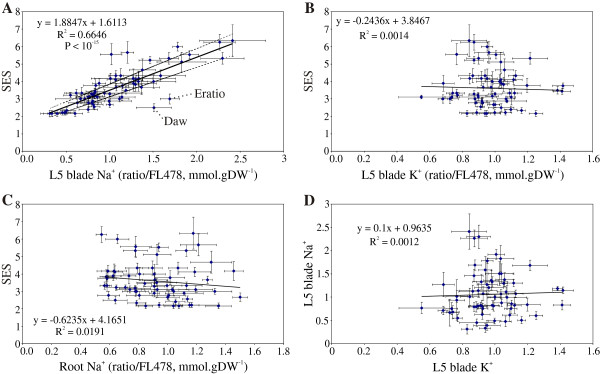
**Salinity-induced injury is highly correlated with leaf Na**^**+ **^**concentrations across the entire species. **(**A**). The visual SES injury score was highly correlated with leaf Na^+^ concentration across all cultivar-groups of *O. sativa*, and in all tested accessions of *O. glaberrima*. The linear regression line is shown, together with ± SE intervals. However, no such relationship was seen with leaf K^+^ concentration (**B**) or root Na^+^ concentration (**C**). Likewise, there was no relationship between leaf Na^+^ and K^+^ concentrations (**D** ratio/FL478, mmol.gDW^-1^ data). Similar relationships were seen in both the youngest and second-youngest expanded leaf (at time of salinisation; L5 and L6 in these data, and the only leaves still photosynthetically active; leaf 6 data not shown). FL478 was used as the tolerant check.

The visual SES scores showed a continuous distribution, highlighting the polygenic nature of salinity tolerance. Na^+^ concentrations also showed a wide range and continuous distribution and, surprisingly, a strong correlation was observed between SES scores and leaf Na^+^ concentration (Figure 3). This was observed for both linear regression based on average values per line (P < 10^-15^ for leaf 5, Figure 3A; also P < 10^-17^ for leaf 6, data not shown) and Spearman’s rho based on observations per plant (P < 10^-58^, Additional file 2: Table S2). This relationship held true among almost all *O. sativa* accessions, held for all leaf blades sampled and for the leaf 6 sheath and also held true for *O. glaberrima*. In contrast, little or no such relationship is seen for Na^+^ concentrations in roots, leaf K^+^ concentration or between Na^+^ and K^+^ concentrations (Figure 3B–D). Significant associations were also observed between SES scores and leaf 5/leaf 6 and the leaf 6 blade/sheath ratios of Na^+^ concentrations (Additional file 3: Figure S1). These showed a much weaker relationship, but appeared to be at least partially independent of the leaf 6 blade Na^+^ concentration and may represent additional tolerance components.

SES scores also showed strong correlations with various biomass parameters (Additional file 2: Table S2). The strongest of these correlations was with leaf 6 sheath biomass (r^2^ = 0.54), followed by total harvested tissue and root biomass (r^2^ = 0.47 and 0.46, respectively). However, correlations with leaf biomass were far lower (r^2^ = 0.14, 0.20 and 0.36 for leaf 4, 5 and 6, respectively). SES scores also correlated significantly with leaf Na^+^ content (as opposed to concentration). The overall correlation was moderate (r^2^ = 0.47), mainly because of a small number of outlying accessions (Additional file 4: Figure S2), all of which carried the *Japonica* or *IR29* alleles of *OsHKT1;5* (see below). Excluding these accessions produced a strong correlation (r^2^ = 0.69).

**Figure 4:**
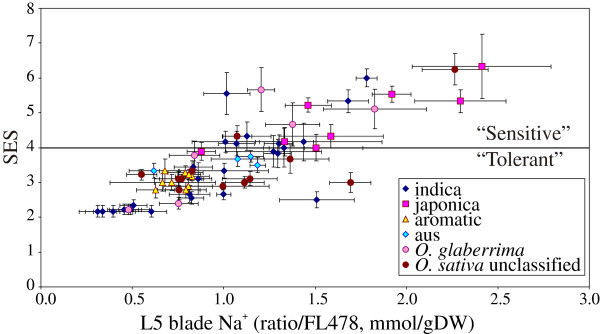
**Tolerance is not well correlated with cultivar-groups.** Tolerance is not well correlated with cultivar-groups in *O. sativa*, though few japonica accessions score tolerant overall, and few aromatic accessions score sensitive. Members of the au*s* cultivar-group generally score moderate to highly tolerant; indica accessions show a wide variability. Few accessions of *O. glaberrima* have been screened, but these seem to show as wide a range of tolerance and Na^+^ exclusion as seen in *O. sativa.*

However, the correlation of SES scores (and Na^+^ concentration) with cultivar-group was not so clear (Figure 4). The aromatic and, to a lesser extent, the aus accessions were all in the “tolerant” class (SES score < 4) despite not being chosen for tolerance, whereas the japonica types were mostly sensitive. Accessions from the indica group and *O. glaberrima* showed a wide range in both tolerance and Na^+^ concentration.

### Association of tolerance with *HKT1;5* allele groups

**Figure 5:**
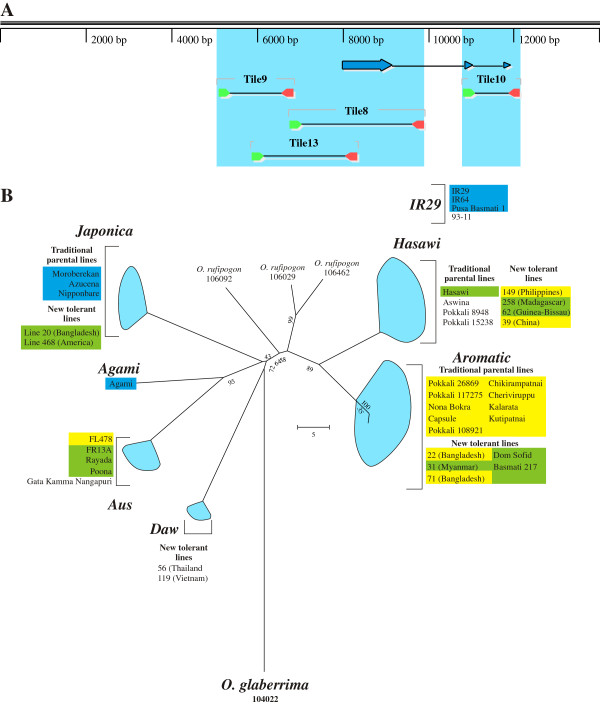
**Allele mining of *HKT1;5 *from *O. sativa *and *O. glaberrima.*** Sequencing and phylogeny of *HKT1;5* from *O. sativa* and *O. glaberrima*. **A**. Regions amplified and sequenced. These total approximately 6.5 kb, including the full coding region and approximately 3.5 kb of promoter. Exons of the *OsHKT1;5* gene are shown as filled, linked arrows, primers/PCR products by linked green and red arrows. **B**. Minimum-evolution tree of sequenced regions, based on the number of differences (10,000 bootstrap replicates, pairwise deletion of gaps). Selected lines possessing each allele are indicated. Yellow shading indicates high tolerance and high Na^+^ exclusion, and green indicates moderate tolerance and exclusion. Blue shading indicates sensitivity and low Na^+^ exclusion. The *Daw* allele lines (unshaded) are tolerant/highly tolerant but do not show the same amount of Na^+^ exclusion. Other unshaded lines have not been tested for salinity tolerance or leaf Na^+^ concentrations.

*HKT1;5* has been identified as a major determinant of tissue Na^+^ concentration and salt tolerance in rice [24] and wheat [26], and circumstantial evidence points to a role also in barley, sorghum and maize (personal observations). Therefore, it was of interest to determine the correlation of salt tolerance and tissue Na^+^ concentration in rice with *HKT1;5* allelic diversity; a related question is to determine whether multiple mechanisms exist for reducing tissue Na^+^ concentration. Portions of the *HKT1;5* gene totalling approximately 6.5 kb, including the entire coding region and about 3.5 kb of promoter, were amplified from selected lines, with a focus on newly identified tolerant lines from diverse cultivar-group/geographic backgrounds. A total of seven major alleles were identified within *O. sativa*, together with three minor alleles within the *Japonica*, *Aromatic* and *IR29* allele groups (Figure 5; minor alleles are not easily visible due to the scale of the tree, but are present in the varieties Azucena, Dom Sofid and IR29, respectively). Interestingly, the allele present in the sensitive line IR29 (and shared with the reference genome of 93–11) is a chimeric allele, with the promoter, transcription and translation start shared with the *Hasawi* allele, fused to the 3^′^ regions (including the remainder of the coding regions) of the *Japonica* allele. This allele to date has been identified only in improved indica-group cultivars, namely, IR29, IR64, Pusa Basmati 1 and 93–11. Chimeric sequences tend to destabilise phylogenetic trees by artificially inflating branch lengths and producing incorrect topologies, and thus the *IR29* sequence has been excluded from the tree shown in Figure 5.

In general the various alleles could be assigned to specific cultivar-groups of rice, based on ubiquitous occurrence in several accessions known to be essentially pure representatives of those cultivar-groups. Thus, the *Japonica*, *Aromatic* and *Aus* alleles are quite easily identified. The *Hasawi* allele (so named because it was first isolated from Hasawi, a tolerant landrace from Saudi Arabia) is found in many accessions, but is overrepresented in those from the indica cultivar-group and it may represent the allele originally from that group. The geographic provenance of accessions carrying the *Japonica* and *Hasawi* alleles is wide-ranging, indeed global (Figure 1). The *Aromatic* allele seems to stem solely from southern Asia (India and Bangladesh) and the northern Middle East (Iran), but nonetheless appears common. The *Aus* allele appears largely restricted to South Asia, notably around eastern India and Bangladesh, as typical for the aus cultivar-group [47] in which it is overrepresented. The *Daw* and *Agami* alleles are exceptionally rare, and do not fit into the generally accepted divisions of *O. sativa*, or seemingly the older rayada and ashina clades [47,48]. The *Daw* allele has been found so far in only two accessions, one from Thailand and the other from Vietnam, both of which are in the indica cultivar-group. The *Agami* allele is so far found only from Agami Mont 1 (IRGC 3084), an Egyptian traditional cultivar reported to be in the japonica cultivar-group and possessing mild salinity tolerance (present data and, e.g., [8,49]). The origin of these alleles is uncertain. They are clearly distinct and separate alleles, not derived from any of the other identified alleles by simple mutation or recombination, and it is tempting to speculate that they may represent either remnants of otherwise now-extinct cultivar-groups or introgressions from wild relatives. Further sequencing (particularly whole-genome sequencing) would be needed to clarify this further.

**Figure 6:**
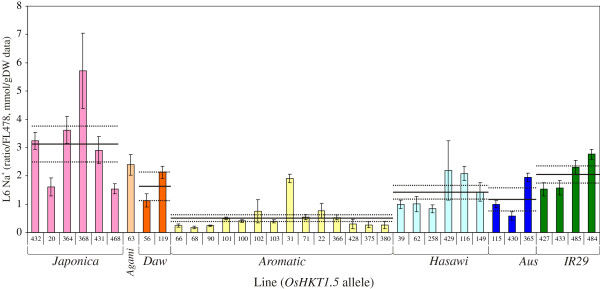
**Na**^**+ **^**concentration in the leaf is highly associated with the *HKT1;5 *allele across diverse accessions.** Association of Na^+^ concentrations in leaves with the *HKT1;5* allele. Mean (solid horizontal line) and SE (broken horizontal lines) for each allele group are indicated. Lines carrying the *Aromatic* allele generally showed the greatest exclusion, followed by the *Aus* and *Hasawi* alleles. Lines carrying the *Japonica* allele generally showed the least exclusion, followed by lines with the *IR29* allele.

Examination of Na^+^ concentrations in representatives of the different allele groups showed a surprisingly strong association between the *HKT1;5* allele and overall tissue Na^+^ concentration (Figure 6). This was seen after 11 days of salt stress in both the youngest and second-youngest expanded leaf (marked at the time of salinisation), though this was clearest in the latter. The *Aromatic* allele group clearly showed the highest exclusion overall. This was followed by the *Aus* and then *Hasawi* allele groups, though the difference between these was not statistically significant. The *IR29*, *Daw* and *Agami* allele groups seem to have approximately the same exclusion though sample sizes were too small to make confident generalisations. The *Japonica* allele group had by far the highest overall Na^+^ concentrations, and even the two newly identified tolerant lines that showed “low” Na^+^ were only the equal of the highest concentrations seen in the *Hasawi* and *Aus* allele groups. Thus, comparison of the average Na^+^ concentrations across a number of diverse landraces allows a tentative hypothesis as to the relative strength of the various alleles:

$$\begin{array}{l} Aromatic>Aus\geq Hasawi\\ \;>Daw≅Agami≅IR29\geq Japonica \end{array}$$

This information should prove useful in breeding programs when choosing the best donor for the *HKT1;5* gene, and it is probably not coincidental that all currently used highly tolerant donors contain the *Aromatic* allele (e.g. Nona Bokra, Pokkali, Cheriviruppu, Kala Rata, Kuti Patnai, Chikiram Patnai, Capsule). However, it is notable that FL478, despite its high tolerance, actually carries the *Aus* allele, which does not seem to be as effective.

### Novel sources and mechanisms of salinity tolerance

As can be seen from Figure 3A and Figure 4, salinity tolerance in rice (as measured by the visual SES score) is highly correlated with leaf Na^+^ concentration in an extremely diverse set of germplasm, encompassing all cultivar-groups and all known *HKT1;5* alleles from *O. sativa* and even including *O. glaberrima*. This is true for both “sensitive” and “tolerant” germplasm. Clearly many of these lines are displaying high (or low) tissue Na^+^ concentrations largely due to the particular *HKT1;5* allele they possess. Thus, the association between tissue Na^+^ concentration and the *Aromatic* and *Japonica* alleles is generally quite strong; however, two accessions carrying the *Japonica* allele (Carolina Gold from Peru and Gachia from Bangladesh; lines 468 and 20 in Figure 6) show reasonably low tissue Na^+^. These therefore probably possess novel mechanisms maintaining low Na^+^ uptake.

**Figure 7:**
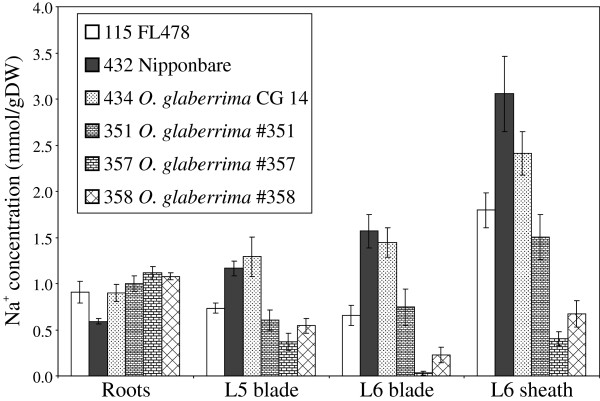
**Na**^**+ **^**concentration in tolerant *O. glaberrima *accessions.** Na^+^ concentrations in tolerant *O. glaberrima* accessions; CG14 is included as a sensitive check. Lines 351, 357 and 358 all showed exclusion equivalent to or better than FL478, the tolerant check; in the case of 357, it was below reliable detection limits in leaf 6.

Likewise, although very little is still known about the situation in *O. glaberrima*, tolerant accessions from this species also show extremely low tissue Na^+^ concentrations (Figure 7) – such that concentrations in the youngest leaf were below the reliable detection limit for one accession (line 357 in Figure 7; IRGC 104022). However, line 357 appears to have the same *HKT1;5* allele as several sensitive lines that show very high Na^+^ concentrations (e.g. CG14, IRGC 103455 and IRGC 104038; see phenotyping data in Table 1, Supplemental Table 1 and Figure 7; the *OgHKT1;5* allele from IRGC 104022 is GenBank accession JQ695813), and is therefore also likely to possess exclusion mechanisms apart from *HKT1;5*.

**Figure 8:**
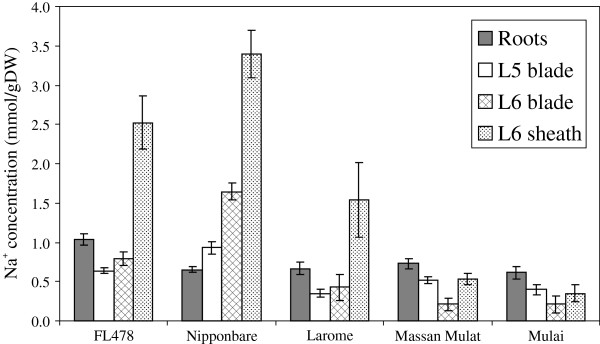
**Na**^**+ **^**concentrations in selected accessions from Iran.** Na^+^ concentrations in various organs of selected accessions from Iran and checks. Note that while FL478 (tolerant check) has lower concentrations in its leaf blade and sheath than a sensitive line such as Nipponbare, it actually contains an increased concentration in roots. This is typical of many tolerant Na^+^-excluding lines, but the relationship is broken in these lines from Iran (Larome, Massan Mulat, Mulai); which contain low Na^+^ concentrations in roots in addition to aerial portions.

A further observation on leaf Na^+^ concentrations is the tendency of many Na^+^-excluding lines to show decreased leaf Na^+^ concentrations, but increased concentrations in roots relative to sensitive lines. This is seen in FL478 and most of the excluding lines described here, including the tolerant *O. glaberrima* accessions. However, a small number of accessions actually display lower Na^+^ concentrations in both roots and leaves (and leaf sheath, Figure 8). Notable among these are Massan Mulat and Mulai from Iran, Carolina Gold from Peru, Rayada from Bangladesh and possibly Eratio from Senegal. The low Na^+^ concentration in all sampled organs suggests that these lines may have a mechanism to limit the amount of Na^+^ that is getting into inner parts of the root (probably the stele in particular) in the first place. Such mechanisms may include re-export of Na^+^ via SOS1, or increased suberisation of the endodermal layer, thus reducing the transpirational bypass flow and passive uptake.

Based on the SES scores, Na^+^ and K^+^ concentrations data examined in these experiments, maintaining low leaf Na^+^ concentration is probably the major mechanism conferring salinity tolerance in *Oryza sativa* and *O. glaberrima*. However, other mechanisms are likely to exist, and two lines of particular interest are Daw Hawm from Thailand (IRGC 44131) and Eratio from Senegal (IRGC 15800). Based on the correlation of SES with leaf 5 Na^+^ concentration, both Daw Hawm and Eratio show much lower SES scores than expected (Figure 3A). For Daw Hawm, this is even more pronounced under 180 mM NaCl (data not shown). The correlation with SES is much as would be expected if these lines were showing high tissue tolerance; further work is clearly needed to define the mechanisms of tolerance operating in these lines.

### Additional evidence for multiple Na^+^ exclusion mechanisms

**Figure 9:**
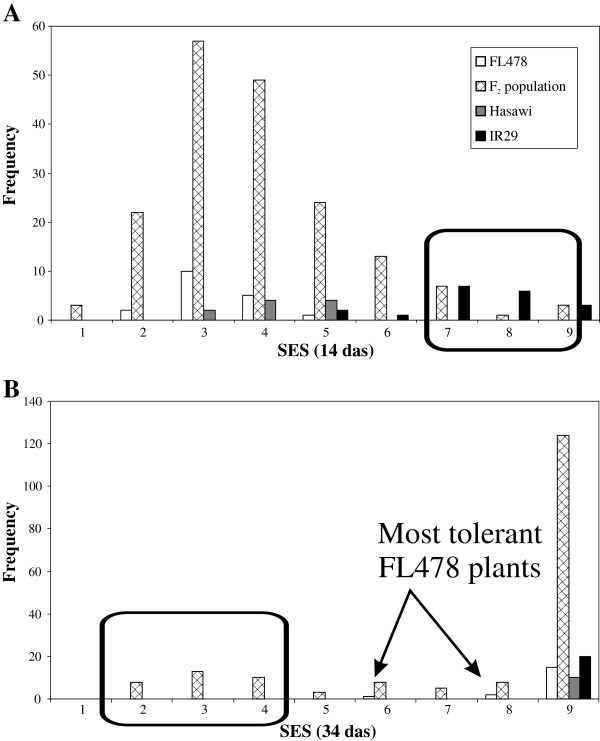
**Genetic separability of tolerance mechanisms.** Further evidence that different lines may have different genes conferring tolerance. SES scores of an F_2_ population derived from the cross of the two tolerant genotypes FL478 × Hasawi were recorded after treatment with 150 mM NaCl (applied at 21 days after germination). The F_2_ population displayed transgressive segregation in both the sensitive (early timepoint, 14 days after salinisation, das; **A**) and tolerant (late timepoint, 34 days after salinisation; **B**) directions, compared with FL478 and Hasawi controls. IR29 (sensitive) is included for comparison.

Examination and comparison of several QTL mapping populations show the presence of multiple QTLs affecting Na^+^ uptake [20,22]; our unpublished data]. These include populations derived from parents such as Pokkali, Nona Bokra, Capsule, Kala Rata, Cheriviruppu and Kuti Patnai. In many of these the *Saltol*/*SKC1* QTL is identified as a major cause and all these examples contain the *Aromatic* allele, but numerous other QTLs of varying effect have been noted. Further genetic evidence for the existence of multiple Na^+^ exclusion mechanisms comes from the examination of SES score distribution in a population derived from a cross between the two tolerant lines, FL478 and Hasawi. Hasawi is a landrace from Saudi Arabia (IRGC16817; G. Gregorio, personal communication, [8]; this may be the same as that reported by [50]) that shows moderate salinity tolerance and leaf Na^+^ concentrations intermediate between FL478 and IR29 (Wei et al. in preparation; present data). SES score distribution in an F_2_ population showed transgressive segregation in both the sensitive and tolerance direction (Figure 9). This indicates that the two parents are likely to have different loci conferring significant tolerance, presumably the mechanisms maintaining low tissue Na^+^ concentrations in these lines, and that these loci segregate independently. Although genetic evidence is yet to be established, similar results could be expected for many of the other lines presented in this study.

## Discussion

Screening of landraces from coastal and saline inland regions identified a number of accessions showing modest to significant salinity tolerance that are distinct from traditionally used donors such as Pokkali and Nona Bokra. These accessions are from diverse backgrounds, including nearly all cultivar-groups of *O. sativa* and also *O. glaberrima*. To our knowledge this is the first report of significant salinity tolerance from *O. glaberrima*, and also from the aromatic cultivar-group of *O. sativa*. Salinity tolerance in rice thus appears to be widespread both geographically and phylogenetically, or, put in another way, tolerance is not well associated with either geographic or cultivar-group origin. Together with the fact that these are landraces and not expected to show relationships apart from gene flow inherent in the species’ history, this suggests that many of these have probably gained tolerance independently and that multiple mechanisms may thus exist.

On the other hand, tolerance is quite well correlated with leaf Na^+^ concentration across almost all accessions of both *O. sativa* and *O. glaberrima*. Despite the diverse origins and relationships of the accessions, tolerance could in almost all cases be explained largely with reference to lower Na^+^ concentrations in the photosynthetically active leaves. This further suggests that processes controlling this are the predominant mechanisms of tolerance in *O. sativa* and *O. glaberrima*, and that tissue tolerance mechanisms (vacuolar sequestration, ROS scavenging, osmotic adjustment, certain hormonal responses) play secondary roles. Na^+^ exclusion from roots, sequestration of Na^+^ in roots, stems and basal portions of the leaf (sheath), partitioning of Na^+^ from leaf to leaf and dilution of Na^+^ content in a large biomass are mechanisms proposed to influence leaf Na^+^ concentration. Na^+^ sequestration is one such mechanism known to operate in a number of species from both the dicots and monocots (e.g., wild and cultivated barleys: [51,52]; durum and bread wheat [53,54]). However, the relationship is not universal. For example, it has not been observed in studies on maize and sorghum ([55-57], although see [58]) and it is an important [54] but not a universal determinant in wheat [45]. In some cases this may be due to a lack of genotypic variability [57]. The fact that total leaf and shoot Na^+^ content (not just concentration) also shows a very strong correlation indicates that Na^+^ sequestration from the leaf blade is a very important contributor to maintaining low tissue Na^+^ concentrations.

Dilution of Na^+^ concentrations through a large biomass is also a well-accepted mechanism for maintaining low tissue Na^+^ concentrations, and Yeo et al. [9] concluded that Na^+^ accumulation (content) showed only a poor correlation with performance in rice, being significantly confounded with plant height; tall varieties showed better tolerance and lower Na^+^ concentrations due simply to dilution of Na^+^ in the larger volume of tissue produced. The data presented provide an apparent contradiction to the latter, but this may be due to the screening conditions: the latter study conducted screening at relatively low salinity (60 mM) for short periods (10 days). Salt concentration of 150 mM NaCl was used for the physiological characterisation presented here, which is higher than that used in most previous screening studies; the higher salt concentration causes a much greater influx of Na^+^, which may overwhelm other mechanisms, notably the effect of plant vigour [9]. Under these conditions, growth effectively ceases in all varieties after the application of the salinity treatment. A few of the most highly tolerant varieties will resume growth after some time, but at a greatly reduced rate; over the lifetime of an experiment, even the most highly tolerant variety will produce only about half a new leaf. This growth arrest actually appears to be an adaptive feature, and lines that try to keep growing show a different type of growth arrest – the youngest leaves expand, but soon yellow and die, presumably due to excessive Na^+^ accumulation. Thus, screening at higher salinity levels may help to reduce the contribution of biomass to tolerance, and so “simplify” the response in this respect.

It is interesting to note that the correlation of SES scores with plant vigour is highest for leaf sheath biomass (r^2^ = 0.54), followed by total harvested tissue and root biomass (r^2^ = 0.47 and 0.46, respectively), but much lower for leaf biomass (r^2^ = 0.14, 0.20 and 0.36 for leaf 4, 5 and 6, respectively). The leaf sheaths and roots are the main tissues known to act as reservoirs for Na^+^ sequestration, such as that mediated by *OsHKT1;5*[24]. Thus, the contribution of biomass may be partly to dilute the Na^+^ taken up, but also to provide a reservoir for sequestration in non-photosynthetic portions.

One gene known to contribute significantly to Na^+^ sequestration in rice and other species is *HKT1;5*[24,26]. Allele mining of this gene revealed seven major allele groups within *O. sativa*, and comparison of leaf Na^+^ concentrations across a number of diverse landraces allows a tentative hypothesis to be proposed as to the relative strength of the various alleles:

$$\begin{array}{l} Aromatic>Aus\geq Hasawi\\ \;>Daw≅Agami≅IR29\geq Japonica \end{array}$$

It should be noted that the most highly active allele, found in traditional donors such as Pokkali, Nona Bokra and others, has almost certainly originated within the aromatic cultivar-group, despite these being indica types. Indeed, although the sample size and fold changes are small, it seems that the most highly tolerant lines are those from the indica cultivar-group that also possess this *Aromatic* allele; these are often more tolerant than lines from the aromatic cultivar-group. It may be that some feature of the indica cultivar-group genetic background is in some way synergistic with the action of the *Aromatic* allele. Alternatively, it has been noted that many aromatic lines (according to the functional definition) have lower salt tolerance due to their inability to produce gamma aminobutyric acid – the same mutation that confers their aromaticity [59]. Although the aromaticity of most of the lines in this study hasn’t been tested, it may be that the *HKT1;5* allele from traditional aromatic lines has evolved higher activity to compensate for this deficiency and, when transferred into other genetic backgrounds, its full effect is seen.

In many cases low tissue Na^+^ concentrations (and therefore tolerance) can be largely explained by the apparent relative activity of the particular *HKT1;5* allele present in a line. This suggests that it is not sufficient to declare a line as a major new donor of tolerance without first determining the *HKT1;5* allele present, and this should be a component of future screening efforts. However, several exceptions do exist and the association of low leaf Na^+^ concentration with the *HKT1;5* allele is not as tight as that for SES score. Examples of these exceptions include accessions such as Carolina Gold (from Peru, tropical japonica cultivar-group, *Japonica* allele of *HKT1;5*), Gachia (Bangladesh, aromatic cultivar-group, *Japonica* allele) and several accessions from the Philippines and China (indica cultivar-group, *Hasawi* allele). These all possess much lower tissue Na^+^ concentrations and higher tolerance than would be predicted from their *HKT1;5* allele. Likewise, tolerant *O. glaberrima* lines showed very low leaf Na^+^ concentrations, yet all share an *OgHKT1;5* allele with several accessions that are manifestly not tolerant and have quite high leaf Na^+^ concentrations (data not shown); thus, it seems likely that these are also using some other mechanisms apart from *OgHKT1;5* that are, nonetheless, highly effective. Also, varieties from Iran and Turkey would fit in this category. Although these mostly possess the *Aromatic* allele of *OsHKT1;5*, they appear to possess an additional mechanism that limits the amount of Na^+^ entering the root (as opposed to reducing the amount of Na^+^ translocated to the shoot) and so, unlike varieties such as Pokkali and FL478, they possess both low shoot and low root Na^+^ concentrations (Figure 8). Thus, in all these cases it seems likely that alternative mechanisms besides *Saltol/OsHKT1;5* (for example, reduced transpirational bypass flow, alternative sequestration mechanisms) are contributing to a reduction in shoot Na^+^ content and concentration.

Thus, while maintaining low tissue Na^+^ concentrations appears to be the predominant trait conferring tolerance in most rice genotypes, the actual mechanisms conferring low tissue Na^+^ concentration may be quite diverse. Genetic evidence from multiple QTL studies (e.g. [20,22]) shows that while *HKT1;5* contributes a major QTL for Na^+^ exclusion, a number of other minor QTLs also exist. The FL478 × Hasawi F_2_ population presented here also suggests that these mechanisms can be alternately separated and combined genetically using molecular markers. Hasawi is a landrace from Saudi Arabia that shows intermediate tolerance and tissue Na^+^ concentrations (Wei et al. in preparation, current data). It is in the *aus* cultivar-group, and is expected to contain QTLs/mechanisms distinct from those found in traditional donors from India and Bangladesh, such as Pokkali, the presumed tolerant donor for FL478 [60,61]. Examination of an F_2_ population derived from these parents showed transgressive segregation in both the tolerant and sensitive directions. This strongly suggests that the mechanisms present in the two parental lines are distinct and can be combined to produce plants with even higher tolerance. Thus, although maintenance of low tissue Na^+^ concentrations appears to be the predominant mechanism of tolerance in *O. sativa* and probably *O. glaberrima*, there appear to be many mechanisms by which this can be achieved, and these mechanisms are possibly additive.

## Conclusions

Despite their reputations as salt-sensitive species, both *O. sativa* and *O. glaberrima* show a wide range of diversity in salinity tolerance. This is well distributed geographically and phylogenetically, yet, at this high salt concentration, tolerance appears to be mostly related to the ability to maintain low Na^+^ concentrations in the most sensitive tissues such as the blades of active leaves. Much of the variation in this trait can be explained in reference to the *HKT1;5* allele, and future screening efforts should include genotyping for this gene to determine the novelty of the germplasm being evaluated. However, despite the uniformity of mechanisms, it appears that multiple highly effective QTLs/genes/pathways are contributing to this tolerance in different accessions, and these genes show at least some, and probably considerable, potential for pyramiding. Furthermore, some lines appear to show evidence of additional mechanisms of tolerance, such as the putative tissue tolerance in Daw Hawm from Thailand and Eratio from Senegal. The number of accessions screened and characterised in this work, although specifically chosen from areas likely to produce tolerant donors, and much larger than examined in similar studies, is nonetheless relatively modest and a more extensive screening may identify additional rare donors that have complementary mechanisms. Thus, there would seem to be considerable scope for additional exploration of genetic resources apart from traditional donors frequently used in breeding, to further improve salinity tolerance of rice and ensure higher productivity of salt-affected marginal soils.

## Methods

### Plant materials

Seeds were obtained from the T. T. Chang Genetic Resources Centre at the International Rice Research Institute, Los Baños, Philippines (http://irri.org/index.php?option=com_k2&view=itemlist&layout=category&task=category&id=573&Itemid=100236&lang=en). Accessions were chosen first based on previous work, both to provide a comparison to previous physiological investigations and to characterise those new tolerance sources for which little or no work was done on them before. Second, passport information on location, breeding status (landrace) and cultural type was examined to find accessions likely to originate from areas that might have been experiencing salt stress. The majority of the accessions were chosen from areas that could reliably be determined as residing within tidal wetlands (mangrove swamps, areas frequently inundated by tidal movements) on the basis of historical information and visual searches on Google Earth. Priority was given to landraces, as many tolerant breeding lines are derived from a small donor pool. Likewise, accessions were chosen to maximise the diversity of their geographic origins, and some accessions representative of the different *O. sativa* cultivar-groups were added from Garris et al. [47] and McNally et al. [48] to maximise the genetic diversity.

Dormancy was broken by incubating seeds at 50°C for 5 days. Seeds were germinated in petri dishes on moist paper towels for 2 to 3 days at 32°C, then transplanted to Styrofoam floats on de-ionised water. The styrofoam floats consisted of 100 holes in a 10 row × 10 column grid. Seedlings were selected for normal growth and transplanted one per hole. After 3 days, seedlings were transferred to Yoshida’s solution [62], adjusted to pH 5.0. The pH of the solution was monitored and adjusted daily with HCl/KOH, and the solution was refreshed weekly. After transplanting, plants were grown in screenhouse facilities with ambient temperature and photoperiod during April-March 2011. After 2 weeks of growth, seedlings were further thinned to six per row to reduce crowding and to ensure uniformity.

### Screening of landraces

Screening was carried out by applying 180 mM NaCl (Sigma-Aldrich, USA) to the hydroponic solution when seedlings reached the 4- to 6-leaf stage (growth stage 2 – 3; [63]). NaCl was applied in 60-mM increments separated by 2 days to reduce osmotic shock. Eighteen entries were screened per Styrofoam float, with FL478 and IR29 included in each tray as tolerant and sensitive checks, respectively. Three plants per entry were retained after the final thinning, with three treatment replicates for a total of nine plants per treatment. Progress of symptoms was monitored and final scoring was done (using the SES, Standard Evaluation System, with 1 denoting normal growth and 9 most plants dead or dying; [63]) once the sensitive check reached an average score of 6 – 7, which was typically 12 – 15 days after the initial salinisation and 9 – 11 days post-180-mM treatment.

### Physiological characterisation

Selected lines from screening experiments were chosen for further physiological characterisation, with particular reference to Na^+^ and K^+^ concentrations. Plants were grown as described for the screening setup, except that stress was carried out at 150 mM NaCl (applied in 75-mM increments) to allow meaningful characterisation of lines whose tolerance is only moderate. After the final SES evaluation at 11 days after salinisation, plants were harvested for determination of ion concentration in the plant tissue. Selected tissues (root, leaf 4, 5 and 6 blade, and leaf 6 sheath) were dissected, washed twice in tap water and twice in de-ionised water, and bagged. Leaves 4, 5 and 6 were chosen as they represented the youngest and most active leaves (leaf 6 the youngest), and it is in these leaves that varietal differences in Na^+^ concentrations are greatest. Samples were dried at 50°C for 5 days and tissue dry weights recorded. Na^+^ and K^+^ were extracted in 0.1 M acetic acid (Sigma-Aldrich, USA, diluted in Nanopure water) at 60°C until fully hydrated and tissue was leached. Na^+^ and K^+^ were measured on a Perkin-Elmer AAnalyst200 atomic absorption spectrophotometer (Perkins Elmer, USA), operating in emission mode. Data manipulation and simple statistical analyses were performed in Microsoft Excel, while Spearman’s correlations were calculated using SPSS v. 13.

### SNP genotyping and allele mining of *HKT1;5*

Leaf tissue was harvested from bulked samples and frozen in liquid N_2_. Samples were ground to a fine powder in liquid N_2_, and DNA extracted with a phenol-chloroform method. DNA was quantified on a NanoDrop 2000 (Thermo Scientific, USA) and diluted to 100 ng/μL. SNP genotyping was carried out by Dr. Michael Thomson, IRRI, on an Illumina BeadExpress system using the 384-plex *indica-indica* assay as described in Thomson et al. [46].

Portions of the *HKT1;5* gene were amplified with Phusion Hotstart II polymerase (Finnzymes, USA) and cloned into the EcoRV site of pZErO2 (Invitrogen, USA). Ligations were transformed into chemically competent XL10-Gold cells (Stratagene, USA). Primers used are described in Table 2.

**Table 2:** **Primers used to amplify *HKT1;5 *from rice**

**Primer**	**Sequence (5′ – 3′)**	**Tm (°C)**	**Size (bp)**
Tile8For	GTCGCCTCCCTCCAGCTAATGTACTGTC	78.7	3102
Tile8Rev	GGCCTCCAACAAACTGAAAGCGTCAAT	79.6	
Tile9For	GGCGGTGGGTGGTGCTTGGGTAGAGATA	83.9	1806
Tile9Rev	GATGACAAGAGCGGCCGACAGTACATTA	78.8	
Tile10For	CTACACTGAATTATACTGCGTGAAC	65.5	1390
Tile10Rev	TAGAGCTCGACCAGATCCTGATATAGAC	71.1	

Primers used to amplify *HKT1;5* from *O. sativa* and *O. glaberrima*.

Positive clones were identified based on blue/white screening with X-Gal (Invitrogen, USA) and confirmed by restriction digests. Sequencing was carried out by Macrogen, Korea. Sequencing results were assembled with Lasergene software (DNAstar, USA), and exported as fasta consensus files. Fasta alignment and phylogeny estimation was carried out with MEGA5 [64]. Sequences were deposited in GenBank [GenBank: JQ695808 – JQ695818].

## Abbreviations

ROS: Reactive oxygen species

## Competing interests

The authors declare they have no competing interests.

## Authors’ contributions

JDP and JAE contributed to the experimental design. JDP chose candidate accessions and performed the experimental setup, harvest and analysis. JAE performed SPSS analysis. JDP and AMI drafted the manuscript. All authors contributed to the conceptualization of the study, read and approved the final manuscript.

## Supplementary Material

Additional file 1: Table S1 Summary of screening data. Summary of accessions of *O. sativa* and *O. glaberrima* screened and their observed responses to salt stress. Click here for file

Additional file 2: Table S2 Pair-wise correlations between measured parameters. Pair-wise correlations between SES, biomass, ion contents and ion concentrations of Na^+^ and K^+^ in different tissues. Click here for file

Additional file 3: Figure S1 Involvement of leaf-to-leaf and leaf-to-blade partitioning. Leaf-to-leaf partitioning and sheath-to-blade partitioning of Na^+^ are both clearly correlated with SES, though the relationship is weaker than that for SES. Both may be at least partially independent of actual L6 Na^+^ concentrations. **A**, SES vs. L6 blade/sheath ratio of Na^+^ concentrations. **B**, SES vs. L6/L5 ratio of Na^+^ concentrations. **C**, L6 Na^+^ concentration vs. L6 blade/sheath ratio of Na^+^ concentrations. **D**, L6 Na^+^ concentration vs. L6/L5 ratio of Na^+^ concentrations. FL478 was the tolerant check. Click here for file

Additional file 4: Figure S2 Correlations of SES with biomass and Na^+^ content (mmol/sample) parameters. Click here for file
