# Supplementary figures and images for: Salinity tolerance, Na+ exclusion and allele mining of HKT1;5 in Oryza sativa and O. glaberrima: many sources, many genes, one mechanism?
Source: BMC Plant Biol. 2013 Feb 27;13:32. doi: 10.1186/1471-2229-13-32 (PMC3599985; doi:10.1186/1471-2229-13-32)

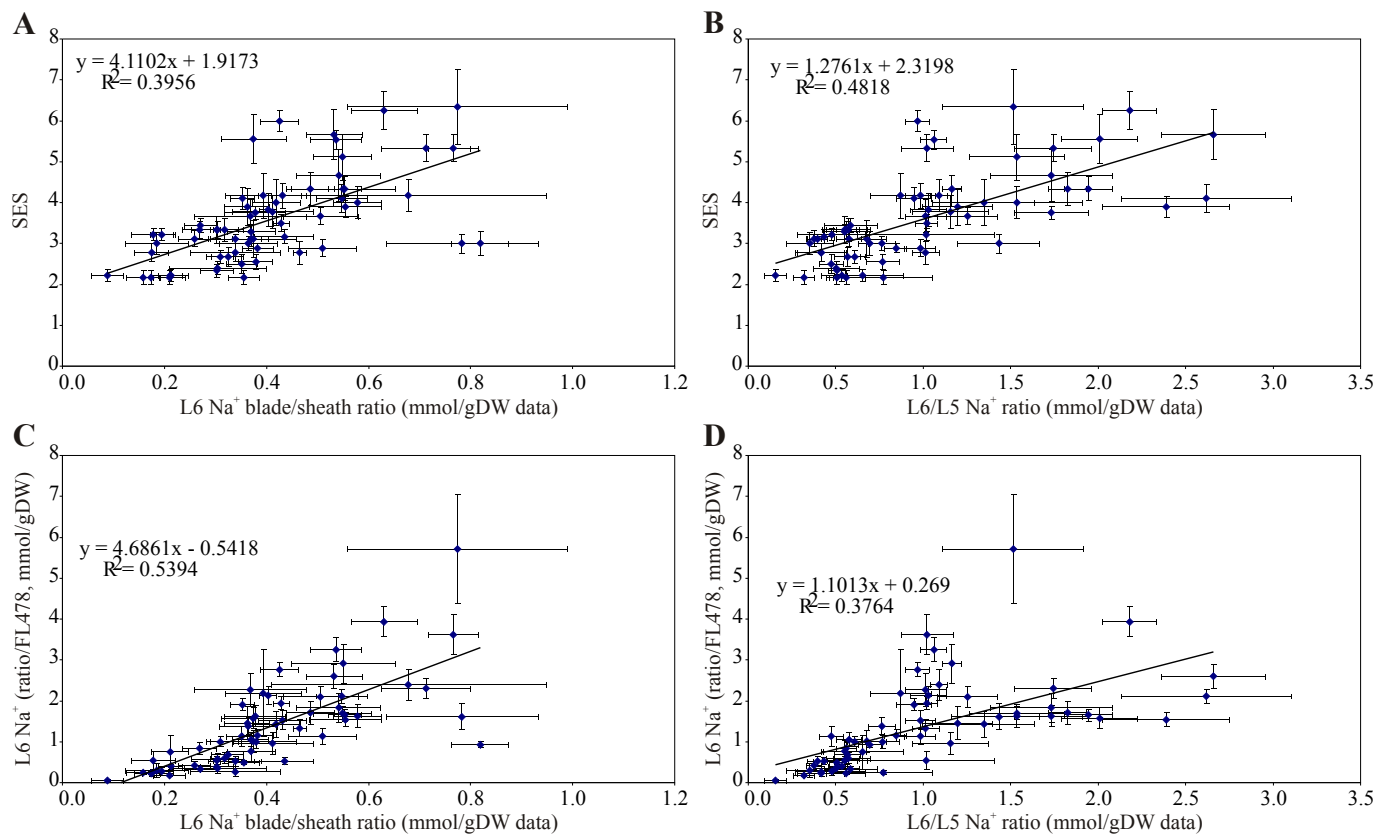

Supplement: Additional file 3: Figure S1 — Involvement of leaf-to-leaf and leaf-to-blade partitioning. Leaf-to-leaf partitioning and sheath-to-blade partitioning of Na+ are both clearly correlated with SES, though the relationship is weaker than that for SES. Both may be at least partially independent of actual L6 Na+ concentrations. A, SES vs. L6 blade/sheath ratio of Na+ concentrations. B, SES vs. L6/L5 ratio of Na+ concentrations. C, L6 Na+ concentration vs. L6 blade/sheath ratio of Na+ concentrations. D, L6 Na+ concentration vs. L6/L5 ratio of Na+ concentrations. FL478 was the tolerant check. [file 1471-2229-13-32-S3.pdf]

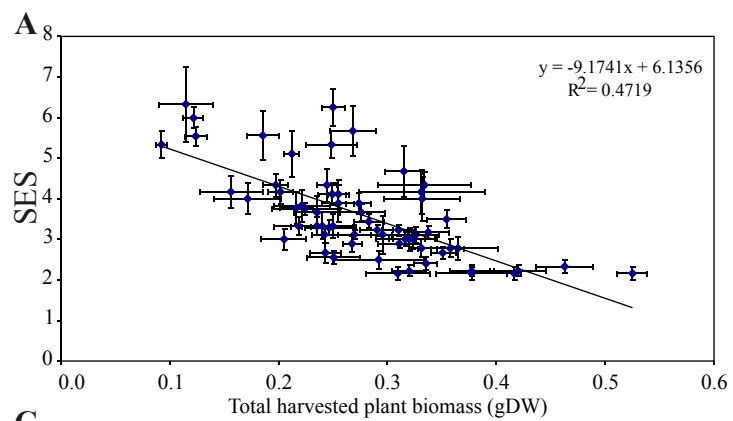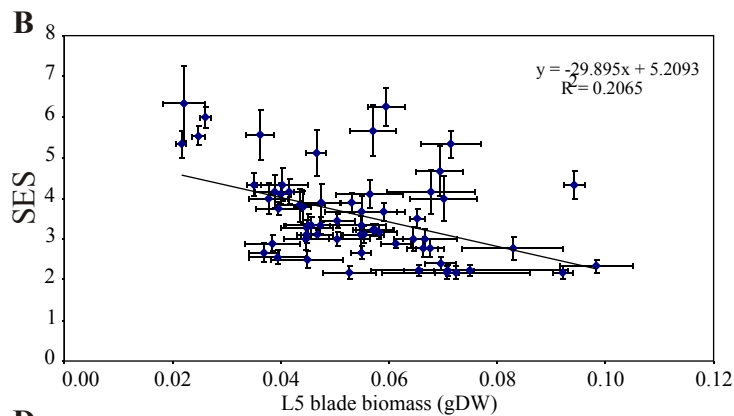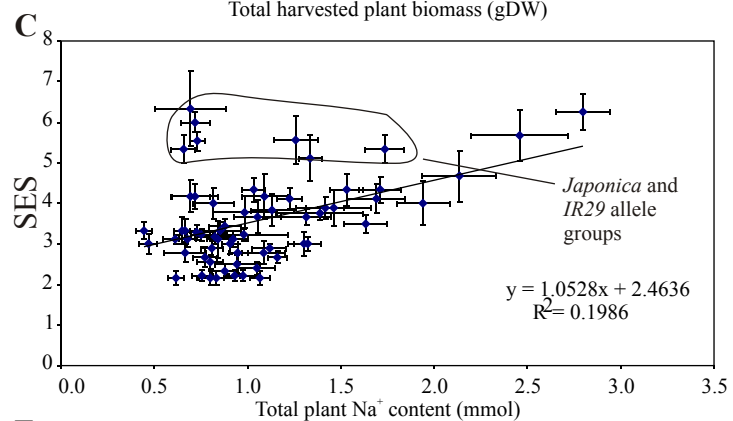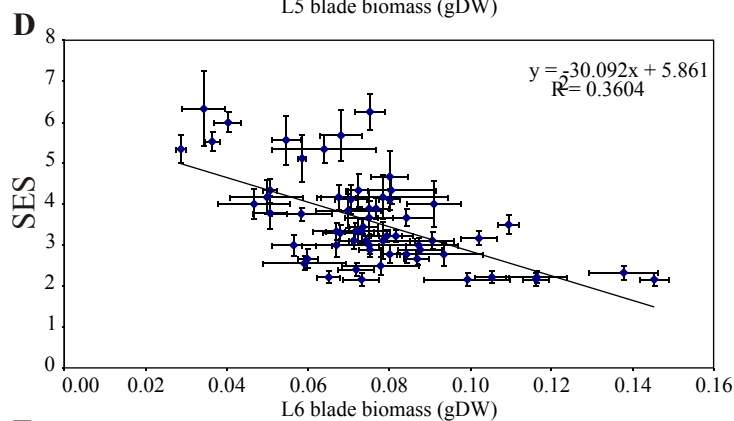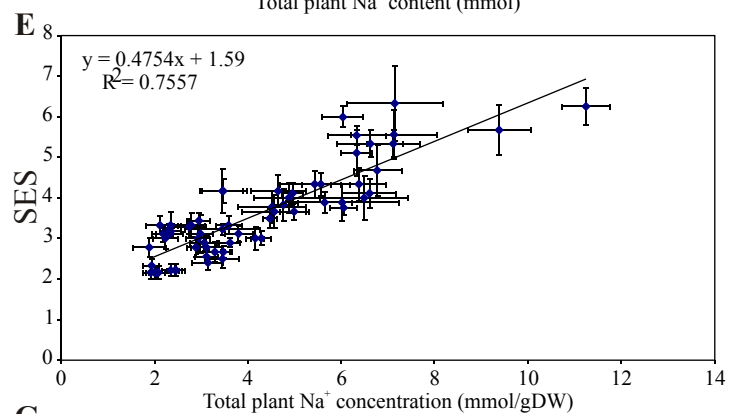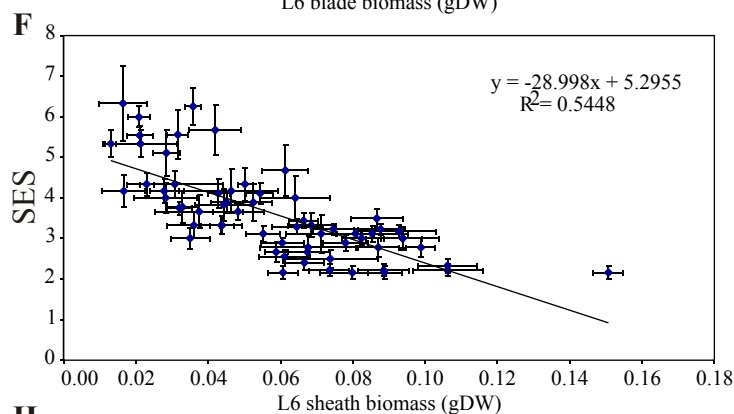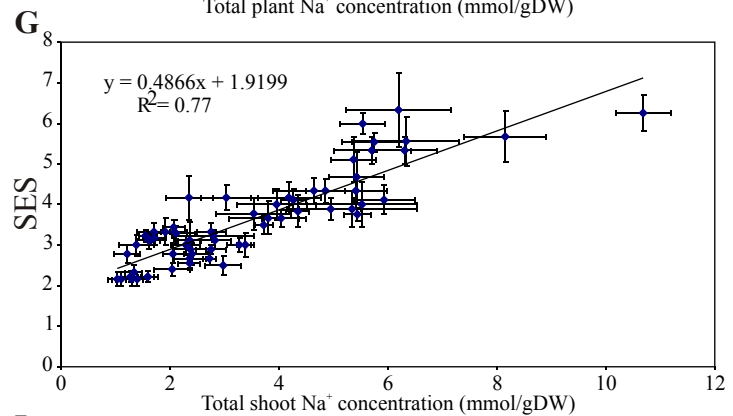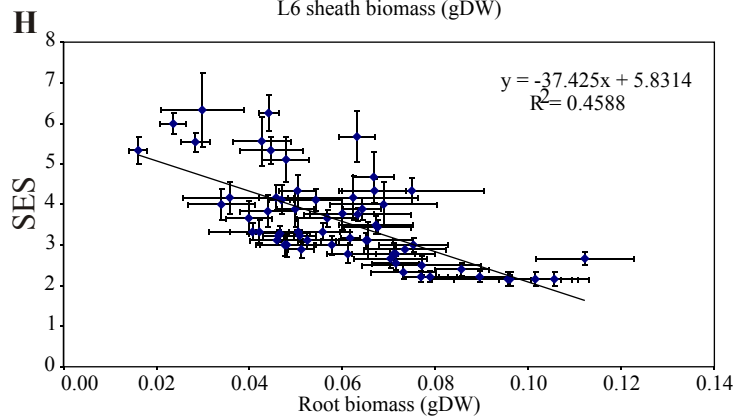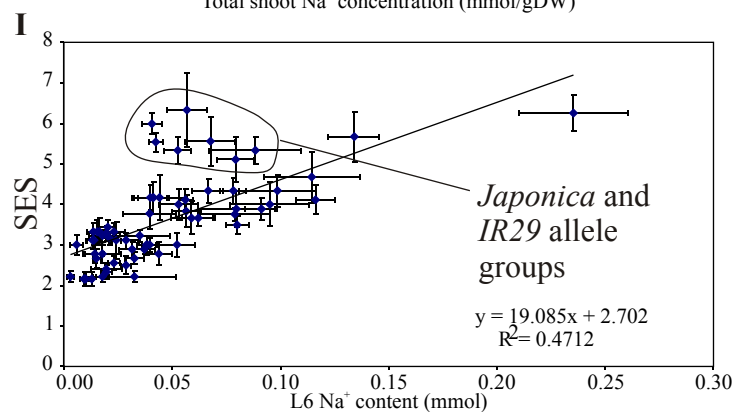

Supplement: Additional file 4: Figure S2 — Correlations of SES with biomass and Na+ content (mmol/sample) parameters. [file 1471-2229-13-32-S4.pdf]
